# Surveillance for incidence and etiology of early-onset neonatal sepsis in Soweto, South Africa

**DOI:** 10.1371/journal.pone.0214077

**Published:** 2019-04-10

**Authors:** Sithembiso C. Velaphi, Matthew Westercamp, Malefu Moleleki, Tracy Pondo, Ziyaad Dangor, Nicole Wolter, Anne von Gottberg, Nong Shang, Alicia Demirjian, Jonas M. Winchell, Maureen H. Diaz, Firdose Nakwa, Grace Okudo, Jeannette Wadula, Clare Cutland, Stephanie J. Schrag, Shabir A. Madhi

**Affiliations:** 1 Department of Pediatrics, School of Clinical Medicine, Faculty of Health Sciences, University of the Witwatersrand, Johannesburg, South Africa; 2 Centers for Disease Control and Prevention, Atlanta, United States of America; 3 Centre for Respiratory Diseases and Meningitis, National Institute for Communicable Diseases of the National Health Laboratory Service (NHLS), and School of Pathology, Faculty of Health Sciences, University of the Witwatersrand, Johannesburg, South Africa; 4 Department of Clinical Microbiology and Infectious Diseases, NHLS, South Africa and School of Pathology, University of the Witwatersrand, Johannesburg, South Africa; 5 Medical Research Council: Respiratory and Meningeal Pathogens Research Unit, Faculty of Health Sciences, University of the Witwatersrand, Johannesburg, South Africa; 6 Department of Science and Technology/National Research Foundation: South African Research Chair Initiative in Vaccine Preventable Diseases, Faculty of Health Sciences, University of the Witwatersrand, Johannesburg, South Africa; University of Florida College of Medicine, UNITED STATES

## Abstract

**Background:**

Globally, over 400,000 neonatal deaths in 2015 were attributed to sepsis, however, the incidence and etiologies of these infections are largely unknown in low-middle income countries. We aimed to determine incidence and etiology of community-acquired early-onset (<72 hours age) sepsis (EOS) using culture and molecular diagnostics.

**Methods:**

This was a prospective observational study, in which we conducted a surveillance for pathogens using a combination of blood culture and a polymerase chain reaction (PCR)-based test. Blood culture was performed on all neonates with suspected EOS. Among the subset fulfilling criteria for protocol-defined EOS, blood and nasopharyngeal (NP) respiratory swabs were tested by quantitative real-time reverse-transcriptase PCR using a Taqman Array Card (TAC) with 15 bacterial and 12 viral targets. Blood and NP samples from 312 healthy newborns were also tested by TAC to estimate background positivity rates. We used variant latent-class methods to attribute etiologies and calculate pathogen-specific proportions and incidence rates.

**Results:**

We enrolled 2,624 neonates with suspected EOS and from these 1,231 newborns met criteria for protocol-defined EOS (incidence- 39.3/1,000 live-births). Using the partially latent-class modelling, only 26.7% cases with protocol-defined EOS had attributable etiology, and the largest pathogen proportion were *Ureaplasma spp*. (5.4%; 95%CI: 3.6–8.0) and group B *Streptococcus* (GBS) (4.8%; 95%CI: 4.1–5.8), and no etiology was attributable for 73.3% of cases. Blood cultures were positive in 99/1,231 (8.0%) with protocol-defined EOS (incidence- 3.2/1,000 live-births). Leading pathogens on blood culture included GBS (35%) and viridans streptococci (24%). *Ureaplasma* spp. was the most common organism identified on TAC among cases with protocol-defined EOS.

**Conclusion:**

Using a combination of blood culture and a PCR-based test the common pathogens isolated in neonates with sepsis were *Ureaplasma* spp. and GBS. Despite documenting higher rates of protocol-defined EOS and using a combination of tests, the etiology for EOS remains elusive.

## Introduction

Sepsis was attributed to cause approximately 400,000 neonatal deaths in 2015 globally, half of which occurred in sub-Saharan Africa where 34.6% to 66.0% of neonatal deaths reportedly occur within first 24 hours of life.[1–3] There are few studies reporting on etiology of sepsis in the first three days of life from sub-Saharan African countries.[4–10] Ascertaining the etiology of neonatal sepsis in resource-limited settings is challenging, including due to births taking place outside healthcare facilities and inadequate laboratory facilities.

Furthermore, the yield from blood culture, the gold standard for determining sepsis etiology might be compromised due to inadequate blood volume sampling in newborns, challenges in timely processing of specimens and antecedent antibiotic therapy prior to sampling. In addition, blood culture excludes identification of pathogens not readily culturable.

In order to address this gap, we established a prospective surveillance for serious probable infections in neonates from a large academic hospital in Soweto, South Africa with the aim to evaluate the etiology and incidence of early-onset (first-three days of life) sepsis (EOS). Investigations included blood culture and molecular diagnostic assay on blood and nasopharyngeal respiratory samples in newborns with protocol-defined community-acquired EOS.

## Materials and methods

### Population and setting

We conducted the Sepsis Aetiology in Neonates in South Africa (SANISA) Study between August 2013 and September 2014 at Chris Hani Baragwanath Academic Hospital (CHBAH), a public hospital in Soweto, South Africa. CHBAH delivers two-thirds of annual births from the surrounding urban, low-to-middle income population of Soweto. The majority of Soweto births outside CHBAH occur in public clinics, and if any of these newborns require further medical management, they are referred to CHBAH. During the study period, CHBAH was the only public hospital admitting sick neonates in Soweto. The protocol in the neonatal unit is that all neonates born or referred with high obstetric risk factors e.g. preterm births, those requiring resuscitation at birth, and those with symptoms suggestive of sepsis have blood cultures done. Approximately 90% of all newborns admitted in the first 3 days of life have blood taken for culture. As part of hospital protocol, admissions to neonatal intensive care unit (NICU) are restricted to those requiring intubation for mechanical ventilation and/ or inotropes and are weighing ≥900 grams. Neonates weighing <900 grams are only offered nasal continuous positive airway pressure if they weigh ≥750 grams.

### Study design and procedures

This was a prospective surveillance, to identify cases of physician-suspected infection in neonates <28 days of age, with the present analysis restricted to those <3 days of age. The primary objective was to characterize the etiology of EOS and determine its incidence rates. Neonates were eligible for inclusion if blood cultures were collected as per standard care for physician-suspected sepsis. Neonates with major congenital abnormalities, care redirected for anticipated demise, or born to mothers <18 years of age were excluded. Due to an inability to enroll patients at all times, we excluded neonates born from 3:00pm Friday to midnight Saturday from numerators and denominators. Clinical data were collected by medical record abstraction performed by trained study staff using a standardized form, which included maternal and neonatal demographics, sepsis risk factors, clinical presentation, care received, laboratory findings, and clinical outcome.

On admission, as per hospital protocol, physicians collected blood for bacterial culture from all neonates with suspected EOS. The standard of care was for lumbar puncture only to be performed in suspected EOS if there were signs suggestive of meningitis. For blood culture, up to 1ml whole blood was placed in pediatric blood culture bottles (Biomerieux, South Africa), and any remaining volume (minimum: 0.5ml) placed in an ethylenediaminetetraacetic acid (EDTA) tube for study-specific molecular testing. After maternal consent was obtained, study staff collected respiratory specimens (nasopharyngeal and oropharyngeal swabs, NP/OP) from neonates with suspected sepsis. The NP/OP swabs were pooled in universal transport media. Blood in EDTA tube and respiratory samples for molecular testing were stored at -20°C until transfer, which was within 24-hours of collection, for storage at -70°C.

### Study definitions

All physician-suspected EOS cases enrolled were further evaluated to establish if they fulfilled criteria of protocol-defined EOS based on a predefined clinical and laboratory algorithm previously used in this population (S1 Table).[11] A case was defined as culture-confirmed EOS if a significant pathogen was grown from either blood or CSF. Cases were defined as severe if admitted to NICU, had lethargy (defined as absent spontaneous movement and/or decreased level of activity despite stimulation), or died.[12, 13] Prematurity was defined as gestational age <37 weeks, and low birthweight as <2,500 grams.

### Laboratory analysis

Blood culture and CSF specimens were processed per National Health Laboratory Services protocol using the BacT/Alert automated culture system (BioMerieux, Marcy l’Etoile, France). If bacterial growth was detected, a Gram-stain was performed and the sample sub-cultured onto appropriate media. Classification of culture isolates as contaminants was similar to that used in the Aetiology of Neonatal Infection in South Asia (ANISA) study.[14] The organisms that were considered contaminants were coagulase-negative *Staphylococcus* spp, *Bacillus* spp, *Micrococcus* spp, *Corynebacterium* spp, *Propionibacterium* spp, and *Diphtheroids*. Study-specific blood and respiratory specimens underwent molecular testing using custom TaqMan Array Cards (TAC) (Thermo Fisher, Waltham, USA) which allowed simultaneous testing of 21 organisms (9 bacteria and 12 viruses) for respiratory specimens and 12 agents (11 bacteria and 1 virus) for blood samples (S2 Table). TAC designs and assays were adopted from the ANISA study and have been validated in previous studies.[15–17] A negative template control and a positive control consisting of combined RNA transcripts were included on each TAC.[18]

Study quality control identified an unexpected increase in blood TAC detection of *Pseudomonas aeruginosa* in samples collected between July and September, 2014 (30%; 70/234) positive); before July: 1% (7/698) positive). Investigation of these results suggested specimen contamination before processing. Therefore, we excluded all blood TAC results for *P*. *aeruginosa* during this 3-month period.

### Assessment of background rates of pathogen-specific TAC positivity

Molecular tests such as TAC often have a background positivity rate in a healthy uninfected population; which may stem from organism carriage, presence of non-viable genetic material, or very low copy numbers of a pathogen. Such background rates typically vary by age, location and season. Because prior information on background positivity rates for the pathogens of interest was not available for Soweto newborns, in parallel to the active surveillance we enrolled 312 newborns at CHBAH without sepsis (a target of 25 per month), obtained blood and respiratory samples for TAC testing.

### Statistical analysis

Incidence was calculated as cases per 1,000 live births. Administrative live birth data from CHBAH and Soweto community clinics were used as population denominators corrected for the excluded 32-hour period each week. Established patient referral protocols limited the chance that neonates living outside the hospital catchment area were admitted to CHBAH, especially within 72-hours of birth. Confidence intervals for observed incidence (i.e., culture-based incidence) were calculated based on a Poisson distribution.

The primary analytic objective was to estimate the proportion of sepsis episodes attributed to each pathogen evaluated in the study, referred to throughout as “pathogen proportions.” Because the study captured two specimen samples (blood and respiratory), conducted up to three tests per pathogen (blood culture, blood TAC, respiratory TAC), and because each of these tests had its own true positive rate (TPR or sensitivity) and false positive rate (FPR or 1-specificity) for detection of the true cause of the sepsis episode, we adapted the partial latent class model (pLCM) methods developed by Wu et al[19, 20] for application to our surveillance as detailed in the text in S1 Text. All protocol defined EOS cases with at least one laboratory test result available were included in the model input dataset, along with blood culture final determination (for neonates with sepsis) and observed binary (positive or negative) results of the TAC tests. When a culture contained multiple pathogens, a single pathogen was assigned based on physician case review. Healthy-neonate TAC results were used to establish initial prior values for pathogen-specific false positive rates. The 312 enrolled healthy-neonates were sufficient to provide initial estimation of false positive rates with a maximum standard error of 3%, and with an even smaller estimation error after model convergence. The pathogen proportions estimated, included the 27 pathogen targets tested by TAC, plus two additional classes: ‘other blood culture’, which included all significant pathogens cultured from blood culture but for which targets were not included on the TAC test (blood or respiratory), and “other/none” for episodes that could not be attributed to one of the evaluated pathogens. The primary output of the model was the population-level pathogen proportions (presented as means with 95% credible intervals); from these individual-level pathogen proportions could also be derived. Covariates included date of enrollment as a continuous variable, maternal HIV-infection status, and severity of infection. Performance of the pLCM model under a range of input dataset conditions was assessed by a series of simulations. Model convergence was assessed through trace and other diagnosis plots. Model assumptions were examined using interim model outputs by stopping the Gibbs sampler at random cycles. Model fit to input data was evaluated by comparing the fitted and observed number of positives for each TAC test and blood culture. Programming and computation were conducted using the R (version 3.2.5, R foundation for statistical computing, Vienna, Austria)), SAS (version 9.3, Cary, NC, USA), and STATA (version 13, College station, Texas, StataCorp LP) platform.

### Ethical considerations

This study was approved by the Human Research Ethics Committee (M120552) of the University of the Witwatersrand, Johannesburg, South Africa and the U.S. CDC through a formal joint review arrangement. The local Ethics review limited enrolment to 320 healthy-newborns, due to considerations of invasive procedure in healthy-newborns without any direct potential for benefit. Written informed maternal consent was obtained for all enrollees.

## Results

### Patient recruitment and descriptive data

**Fig 1:**
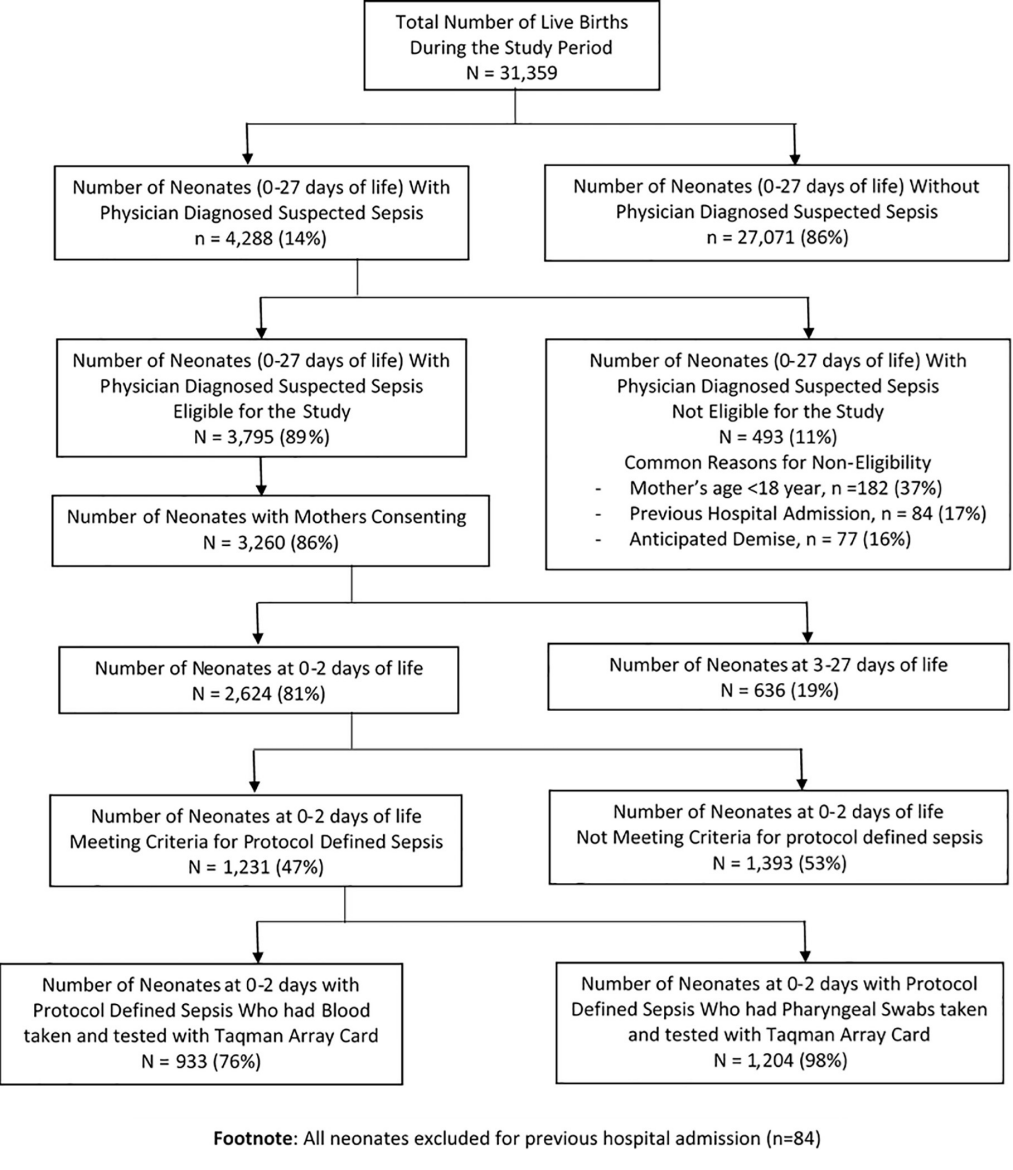
Flow chart of enrolled neonates with physician diagnosed suspected and protocol defined sepsis.

A total of 31,359 live births were documented in public health facilities in Soweto during the study period; of which 4,288 (14%) neonates (0–27 days) were hospitalized for physician diagnosed suspected sepsis, and of whom 3,795 (89%) were eligible for participation. The most frequent reason for exclusion (11% excluded; 493/4,288), was mother’s age <18 years (37%; 182/493), previous hospital admission (17%; 84/493) and anticipated imminent demise (16%; 77/493). Maternal consent was obtained for 86% of eligible cases; including 2,624 (81%) <3 days age, of whom 1,231 (47%) met our protocol-defined EOS definition (i.e. cases) (Fig 1).

Overall, among protocol-defined EOS cases, 55% were male, 56% had low birth weight, 47% born prematurely and 32% born to HIV-infected women. The most common signs of sepsis included tachypnea (32%), metabolic acidosis (36%), lethargy (15%), and hypothermia (15%); whilst fever was uncommon (0.6%). A total of 332 (27%) cases were categorized as severe. The majority of cases were enrolled on day 0 (84%) (Table 1).

**Table 1:** Characteristics of infants meeting case definition* for protocol-defined early-onset sepsis (n = 1,231)—SANISA Study: Soweto, South Africa.

	Neonates with Protocol Defined Sepsis	
**Characteristic**	**Numbers**	**Percent**
**Maternal characteristics**		
Mother: At least one prenatal visit	1136	92.3
Mother: HIV positive	389	31.6
HIV status available**	1175	95.5
**Intrapartum characteristics**		
Delivery: cesarean section	664	53.9
Antibiotics received during labor / delivery	103	8.4
Prolonged rupture of membranes	60	4.9
Meconium stained liquor	277	22.5
**Infant characteristics**		
Male sex	672	54.6
Birth weight categories (n = 1230)		
<1000 grams	114	9.3
1000–1499 grams	222	18.0
1500–1999 grams	195	15.8
2000–2499 grams	155	12.6
≥2500 grams	544	44.2
Gestational age (n = 1218)		
<28 weeks	83	6.7
28–32 weeks	305	24.8
33–36 weeks	181	14.7
≥37 weeks	649	52.7
**Day of life enrolled**		
Day 0 (date of birth)	1035	84.1
Day 1	192	15.6
Day 2	4	<1
**Sepsis signs**		
Poor feeding	4	<1
Lethargy	183	14.9
Hypothermia (T<36° C)	182	14.8
Hyperthermia (>38° C)	7	<1
Hypoglycemia	163	13.2
Hyperglycemia	115	9.3
Metabolic acidosis	441	35.8
Tachypnea (respiratory rate > 60/minute)	398	32.3
**NICU admission**	88	7.2
**Length of stay†: median days (range)**	7 (0–182)	-
**Death††**	149	12.1
**Infection categorized as severe‡**	332	27.0

* Case definition includes clinical and laboratory criteria based on Cutland, et al.[11]

** Infants born to mothers with unknown HIV status were included as no known HIV exposure

† Restricted to infants with known discharge status (alive or died); n = 1173

†† Excludes transferred patients; n = 1193

‡ Severe: infant with noted lethargy, NICU admission, or infection resulted in death

### Culture results

All cases had a blood culture, the mean volume of which was 1.0ml (range: 0.10–1.6ml) per culture (available for 858 cases). Eleven cases received antibiotics before blood collection and a lumbar puncture was performed in 62 cases (5%). Overall, 4.2% (52/1,231) of blood cultures yielded contaminants, 8.0% (99/1,231) yielded any pathogen, of which 7% (7/99) were polymicrobial. The majority of pathogens cultured were Gram-positive (71%), including Group B *streptococcus* (GBS) (35%) followed by *Streptococcus viridans* (24%), and *Enterococcus* spp. (8%); Table 2. The most common Gram-negative pathogens were *Escherichia coli* (8%) and *Acinetobacter* spp. (4%). Three (5%) CSF cultures had a pathogen isolated, with *Acinetobacter* spp., GBS (also positive on blood), and one mixed sample of *Enterococcus* spp. and *S*. *aureus* identified.

**Table 2:** Blood culture isolates by pathogen among neonates with protocol defined early-onset sepsis* (n = 1,231)—SANISA Study, Soweto, South Africa.

Pathogen**	Number of Isolates	Percent
**Gram Positives**	**70**	**70.7**
Group B *Streptococcus*	35	35.4
Viridans streptococci	24	24.2
*Enterococcus* spp.	8	8.1
*Staphylococcus aureus*	3	3.0
**Gram Negatives**	**25**	**25.2**
*Escherichia coli*	8	8.1
*Acinetobacter baumannii*	4	4.0
*Haemophilus influenza*	3	3.0
*Neisseria meningitidis*	2	2.0
*Sphingomonas paucimobilis*	2	2.0
*Enterobacter cloacae*	1	1.0
*Klebsiella pneumoniae*	1	1.0
*Morganella morganii*	1	1.0
*Proteus mirabilis*	1	1.0
*Pseudomonas aeruginosa*	1	1.0
*Salmonella* spp.	1	1.0
**Other Pathogens**	**4**	**4.0**
*Candida* spp. (fungal)	3	3.0
*Mucor* spp. (fungal)	1	1.0
***TOTAL***	**99**	**100**

The following were considered: Coagulase-negative *Staphylococcus* (CONS), Diphtheroids, and *Bacillus* spp. Total blood culture pathogens isolated: 99/1231 (8.0%); Total blood culture contaminants isolated: 52/1231 (4.2%)

*Case definition includes clinical and laboratory criteria based on Cutland, et al.[11]

** Polymicrobial: Cultures with ≥2 pathogens isolated had a single pathogen assigned based on physician case review [7/99 (7%)neonates with positive culture had polymicrobial cultures]

### Molecular diagnostic results

Among cases, 37.3% of blood samples (348/933) tested positive for at least one target and 11.0% with multiple targets. The most common organism detected was *Streptococcus pneumoniae* (14.1%) followed by *Ureaplasma* spp. (9.2%). Among cases respiratory samples, 44.4% (535/1204) tested positive for at least one target and 14.8% for multiple targets. The most common organism detected was *Ureaplasma* spp. (19.9%), followed by *Klebsiella pneumoniae* (11.9%); (Table 3; stratified by case severity and maternal HIV-status in S3 Table and S4 Table respectively). There was poor concordance between blood TAC and cultures for all organisms, with level of concordance varying from 0% for most pathogens to 27.5% for GBS (Table 4).

**Table 3:** Pathogens detected using Taqman Array Card among neonates with protocol defined sepsis* who had blood (N = 933) and respiratory specimens (N = 1204) collected.

	Number of neonates tested positive for different organisms	
**Organism tested in Blood Specimens**	**n**	**%**
*Ureaplasma* spp.	86	9.2
Group B *Streptococcus*	65	7.0
*Staphylococcus aureus*	12	1.3
*E*. *coli/Shigella*	24	2.6
*Streptococcus pneumoniae*	132	14.1
*Klebsiella pneumoniae*	35	3.8
*Salmonella* spp.	29	3.1
*Neisseria meningitidis*	10	1.1
Enterovirus	2	0.2
*Pseudomonas aeruginosa*^†^	7	1.0
Group A *Streptococcus*	2	0.2
*Haemophilus influenza*	6	0.6
***All***	**346**	**37.1**
**Organisms tested in Respiratory Specimens**	**n**	**%**
*Ureaplasma* spp.	240	19.9
Group B *Streptococcus*	102	8.5
*E*. *coli/Shigella* spp.	105	8.7
Enterovirus	17	1.4
Human metapneumovirus	5	0.4
*Klebsiella pneumoniae*	143	11.9
Human parechovirus	4	0.3
Cytomegalovirus	69	5.7
*Bordetella pertussis*	6	0.5
Rhinovirus	8	0.7
*Streptococcus pneumoniae*	27	2.2
Respiratory syncytial virus	3	0.2
Adenovirus	0	0.0
*Chlamydia pneumoniae*	2	0.2
*Chlamydia trachomatis*	6	0.5
Influenza A	0	0.0
Influenza B	0	0.0
*Mycoplasma pneumoniae*	0	0.0
Rubella virus	11	0.9
Parainfluenza virus 1	4	0.3
Parainfluenza virus 2	0	0.0
Parainfluenza virus 3	0	0.0
**All**	**531**	**44.1**

† Total case samples tested for *Pseudomonas aeruginosa = 701*

* Case definition includes clinical and laboratory criteria based on Cutland, et al.[11]

**Table 4:** Concordance between blood culture and Taqman Array Card (TAC) for detecting bacteria in neonates with early-onset sepsis^†^.

	Number with TAC or Culture Positive	TAC and Culture Positive n (%)	Only TAC Positive n (%)	Only Culture Positive n (%)
*Escherichia coli*	21	2 (9.5)	19 (90.5)	0
Group A streptococcus	1	0	1 (100)	0
Group B streptococcus	69	19 (27.5)	46 (66.7)	4 (5.8)
*Haemophilus influenza*	7	1 (14.3)	5 (71.4)	1 (14.3)
*Klebsiella pneumoniae*	36	0	35 (97.2)	1 (2.8)
*Neisseria meningitidis*	5	0	5 (100)	0
*Pseudomonas aeruginosa*	8	0	7 (87.5)	1 (12.5)
*Salmonella* species	29	0	29 (100)	0
*Staphylococcus aureus*	12	1 (8.3)	11 (91.7)	0
*Streptococcus pneumoniae*	132	0	132 (0)	0

^**†**^ Analysis limited to cases in which blood TAC and culture were done (N = 933)

### Background positivity rates among newborns without sepsis

A total of 382 newborns 0–2 days of age without sepsis were screened for participation, 312 (82%) of whom consented. Among these, 20.1% of blood samples (61/304) tested positive for at least one target and 2.6% for more than one target. The most common organism detected was *Streptococcus pneumoniae* (9.9%) followed by *Klebsiella pneumoniae* (2.6%). Among respiratory samples, 54.8% (166/303) tested positive for at least one target and 19.5% for more than one target. The most common pathogen detected was *E*.*coli/Shigella* (19.5%) followed by *Ureaplasma* spp. (14.5%) (Table 5).

**Table 5:** Pathogen detection using Taqman Array Card among healthy infants who had blood (n = 304) and respiratory (n = 303) specimens collected.

	Number of healthy infants with positive test for different organisms	
**Organisms tested in Blood Specimens**	**N**	**%**
*Ureaplasma* spp.	6	2.0
Group B *Streptococcus*	5	1.6
*Staphylococcus aureus*	1	0.3
*E*. *coli/Shigella*	4	1.3
*Streptococcus pneumoniae*	30	9.9
*Klebsiella pneumoniae*	8	2.6
*Salmonella* spp.	7	2.3
*Neisseria meningitidis*	3	1.0
Enterovirus	1	0.3
*Pseudomonas aeruginosa*	6	2.0
Group A *Streptococcus*	0	0.0
*Haemophilus influenza*	0	0.0
***All***	**61**	**19.5**
**Organisms tested in Respiratory Specimens**	**n**	**%**
*Ureaplasma* spp.	44	14.5
Group B *Streptococcus*	40	13.2
*E*. *coli/Shigella* spp.	59	19.5
Enterovirus	26	8.6
Human metapneumovirus	1	0.3
*Klebsiella pneumoniae*	31	10.2
Human parechovirus	1	0.3
Cytomegalovirus	18	5.9
*Bordetella pertussis*	2	0.7
Rhinovirus	3	1.0
*Streptococcus pneumoniae*	15	5.0
Respiratory syncytial virus	3	1.0
Adenovirus	0	0.0
*Chlamydia pneumoniae*	0	0.0
*Chlamydia trachomatis*	0	0.0
Influenza A	0	0.0
Influenza B	0	0.0
*Mycoplasma pneumoniae*	0	0.0
Rubella virus	0	0.0
Parainfluenza virus 1	0	0.0
Parainfluenza virus 2	0	0.0
Parainfluenza virus 3	0	0.0
**All**	**161**	**53.1**

### Etiologic attribution

**Fig 2:**
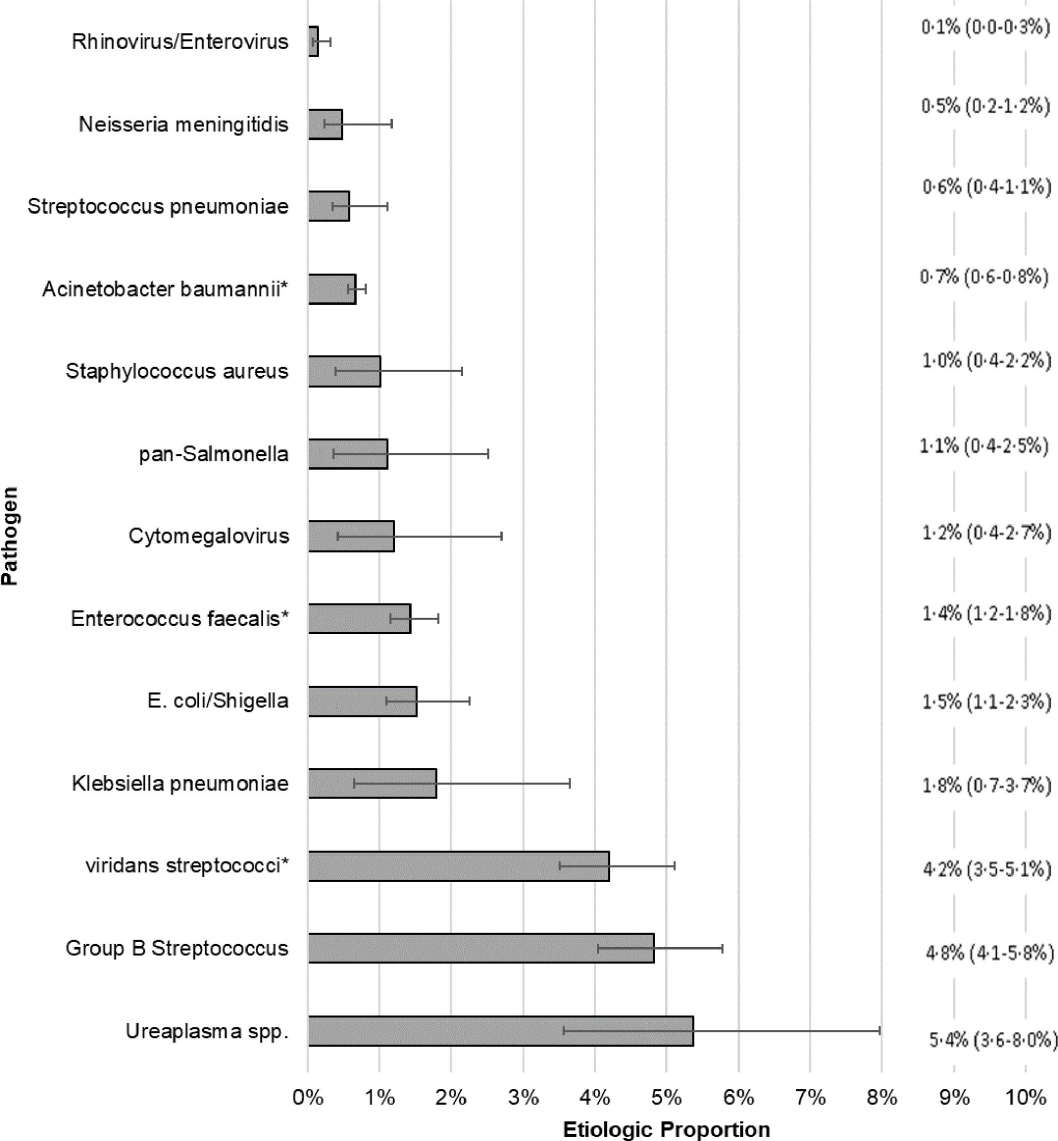
Estimated proportion of total cases caused by each pathogen with 95% confidence interval; Legend: *Pathogen proportion estimated indirectly from the “other blood culture” class. Footnote: 73.3% (68.2–77.2%) of case episodes could not be attributed to an evaluated pathogen class.

Of the 27 organisms considered, 10 were detected at a rate sufficient for etiologic modeling. Estimated TPR by organism and testing method and average FPR for TAC tests are detailed in Table 6. Overall, we were able to attribute etiology for 26.7% (95%CI: 22.8–31.8%) of cases. The three most commonly attributed pathogens being *Ureaplasma* (5.4%), GBS (4.8%), and *Klebsiella pneumoniae* (1.8%), which accounted for 70.0% of the attributable proportion; Fig 2. Individual case-level probabilities for *Ureaplasma* (median probability = 8.2%) were low compared to GBS (median probability = 24.5%). Pathogens in the “other culture” class accounted for an additional 8.7% of the attributable proportion with *Streptococcus viridans* (4.2%), *Enterococcus faecalis* (1.4%), and *Acinetobacter baumannii* (0.7%) being the most common; Fig 2.

**Table 6:** Estimated true positive rate (TPR) and average false positive rate (FPR) in percentages by pathogen and testing method.

	True Positive Rate									Average* False Positive Rate					
	Blood Culture**			Blood Taqman Array Card			Respiratory Taqman Array Card			Blood Taqman Array Card			Respiratory Taqman Array Card		
Pathogen	Mean	2.5Q	97.5Q	Mean	2.5Q	97.5Q	Mean	2.5Q	97.5Q	Mean	2.5Q	97.5Q	Mean	2.5Q	97.5Q
Cytomegalovirus	NA	NA	NA	NA	NA	NA	75.40	41.86	99.93	NA	NA	NA	5.25	4.42	5.84
*E*. *coli/Shigella*	46.30	20.47	78.06	57.14	25.29	89.19	52.98	40.39	75.12	1.00	0.72	1.24	10.31	9.86	10.72
Rhinovirus/Enterovirus	NA	NA	NA	63.05	21.54	99.91	73.19	41.41	99.89	0.32	0.17	0.42	4.23	3.97	4.44
Group B *Streptococcus*	59.57	43.88	74.99	85.80	71.93	95.86	82.48	69.14	93.02	2.26	1.69	2.77	7.38	6.89	7.84
*Klebsiella pneumoniae*	8.54	0.52	30.92	43.81	20.82	83.12	56.44	40.42	95.53	2.29	1.79	2.73	10.69	9.77	11.29
*Neisseria meningitidis*	49.54	6.57	99.80	39.17	20.70	77.29	NA	NA	NA	0.49	0.28	0.62	NA	NA	NA
*Staphylococcus aureus*	32.51	5.94	94.38	60.61	23.87	95.42	NA	NA	NA	0.41	0.16	0.84	NA	NA	NA
pan-*Salmonella*	14.64	0.94	55.13	72.56	25.93	99.96	NA	NA	NA	2.35	1.63	2.82	NA	NA	NA
*Streptococcus pneumoniae*	14.87	0.02	85.09	72.58	36.28	99.93	62.79	40.53	99.62	12.37	11.83	12.85	3.46	3.11	3.74
*Ureaplasma* spp.	NA	NA	NA	87.87	58.86	99.98	79.00	59.86	99.24	2.38	1.45	3.28	13.72	12.48	14.54

True positive rates (TPR): The probability of the test having a positive test result if the case is caused by the pathogen

False positive rates (FPR): The probability of the test having a positive result either for a case, if the pathogen is not the cause, or for a control infant

2.5Q: 2.5% quantile; 97.5Q: 97.5% quantile

* Averaged across modeled covariates (case severity and maternal HIV status)

**TPR for blood culture is equivalent to a pathogen specific estimate of blood culture sensitivity

The observed incidence (per 1,000 live births) of protocol-defined EOS was 39.3 with 12.1% case fatality risk; and the incidence of culture-confirmed EOS was 3.2 with 17.2% (17/99) case fatality risk. Using a combination of tests, the incidence of sepsis attributed to bacteria was 9.7 (95%CI: 8.2–11.5) with Gram-positive incidence (4.7; 95%CI: 4.0–5.7) approximately twice that of Gram-negative (2.8; 95%CI: 2.1–3.8). Overall and stratified by HIV-exposure, *Ureaplasma*, GBS, and *Streptococci viridans* all had an estimated incidence >1 per 1,000 live births (Table 7).

**Table 7:** Observed and estimated pathogen specific incidences per 1,000 live births (with 95% confidence limits) of sepsis* overall and by HIV exposure.

Pathogen	Observed Incidence	Estimated Overall Incidence N = 1,231	Estimated Incidence (HIV Exposed) N = 389	Estimated Incidence (HIV unexposed) N = 842
*Ureaplasma* spp.	NA	2.11 (1.4–3.1)	2.38 (1.3–4.1)	2.01 (1.4–3.0)
Group B *Streptococcus*	1.12	1.90 (1.6–2.3)	1.26 (0.9–1.8)	2.13 (1.8–2.5)
Viridans streptococci	0.70	1.65 (1.4–2.0)	1.93 (1.5–2.6)	1.55 (1.2–2.0)
*K*. *pneumoniae*	0.03	0.70 (0.3–1.4)	0.95 (0.2–2.1)	0.61 (0.2–1.5)
*E*. *coli/Shigella* spp.	0.26	0.60 (0.4–0.9)	0.93 (0.7–1.4)	0.47 (0.3–0.8)
*E*. *faecalis*	0.26	0.57 (0.5–0.7)	0.45 (0.3–0.6)	0.61 (0.5–0.8)
Cytomegalovirus	NA	0.47 (0.2–1.1)	0.58 (0.1–1.9)	0.43 (0.1–1.0)
*Salmonella* spp.	0.03	0.44 (0.2–1.0)	0.95 (0.3–2.0)	0.25 (0.1–0.8)
*A*. *baumannii*	0.13	0.26 (0.2–0.3)	0.28 (0.2–0.4)	0.26 (0.2–0.3)
*S*. *pneumoniae*	—	0.23 (0.1–0.4)	0.38 (0.2–0.8)	0.17 (0.1–0.4)
*N*. *meningitidis*	0.06	0.19 (0.1–0.5)	—	0.26 (0.1–0.6)
*S*. *aureus*	0.10	0.07 (0.03–0.2)	—	0.55 (0.2–1.2)
Rhinovirus/Enterovirus	NA	0.06 (0.03–0.1)	—	0.08 (0.0–0.2)

*Case definition includes clinical and laboratory criteria based on Cutland, et al.[11]

Observed incidence based on pathogen isolation from blood culture specimens

Estimated incidence based on the Partially Latent Class Model developed by Wu, et al.[19]

The proportion of cases attributed to an etiology did not differ by HIV-exposure (HIV-exposed: 26.9%; 95%CI: 21.4–34.5%; no HIV-exposure: 26.7%; 95%CI: 22.1–31.9%). Estimated pathogen proportions were also similar between HIV-exposure groups with the exception of GBS, which was more common in those with no HIV-exposure (5.8%; 95%CI: 4.8–6.9) than HIV-exposed cases (2.8%; 95%CI 2.1–4.0%) (S5 Table).

The proportion of cases attributed to an etiology did not differ significantly between severe and non-severe cases (severe: 31.3%; 95%CI: 26.1–37.4%; non-severe: 26.8%; 95%CI: 22.8–31.8%). However, the pathogen proportion attributed to GBS and *Acinetobacter baumannii* were significantly higher among severe compared to non-severe cases (S1 Fig). Due to previously observed associations between *Ureaplasma* colonization and prematurity,[21] we modeled etiology limited to infants born at 30+ weeks gestation with no substantive change in results.

## Discussion

The high incidence of protocol-defined EOS (39.3 per 1,000 live births) in our study is lower than reported in Asia (55.8 per 1,000 live births).[22] We could not compare these findings with studies from Africa, as we could not find studies that used similar definition of sepsis, and this highlights the need for establishing a harmonized case definition for future epidemiological studies on neonatal sepsis.[23] The incidence of culture proven sepsis in this study was 3.2/ 1000 live births, and the most common organisms isolated were Gram positives, similar to that reported from other sub-Saharan African countries though the common organism was GBS instead of being *Staphylococcus aureus*.[5, 8, 9] This difference could be due to this study mainly enrolling babies born in healthcare-facilities and 84% of cases being investigated within 24 hours of birth. In contrast, up to 50% of births in other African countries are born outside healthcare-facilities.[6, 9] Gram negatives accounted for only 25% of EOS overall, with *Acinetobacter* isolated only in 4%, contrary to that reported in India where *Acinetobacter* (27%), *Klebsiella* (16%) and *Escherichia coli* (14%) accounted for majority of cases.[24]

To address the possibly low sensitivity of blood culture for diagnosing neonatal sepsis, we also investigated suspected neonatal cases using molecular-based assays. The molecular based results, together with blood-culture attributed a putative pathogen to 26.7% of the protocol defined EOS sepsis cases compared to 8% based only on blood culture. Nevertheless, no pathogen was attributed to a high percentage (73.3%) of EOS cases. This could be due to the causative agent not being identifiable on blood culture or not included for detection on the PCR assay. Also, it is possible that the algorithm used was not specific for diagnosing EOS, highlighting the need for better tools by which to diagnose neonatal sepsis. A challenge of using the molecular assay for identifying pathogens, is the background positivity in healthy neonates for some of organisms included in the assay. The partial latent class etiology attribution model used in our study represents a new analytic approach, to interalia address this challenge. This analytical approach has also been applied in large studies of disease etiology[22, 25], and is seemingly pertinent when using molecular diagnostic tools which might not necessarily infer causality, and when using specimens from multiple sampling sites.[22, 26] Although our case definition might still have included some cases that were not truly neonatal sepsis, contributing to the modest attributable etiology, this is inherent to all studies on neonatal sepsis in the absence of a specific and sensitive gold standard clinical definition. Nevertheless, the molecular and analytic methods, including adjusting for “false positive” by inclusion of healthy-newborns, represent a huge advance with an increase in proportion of cases in whom an etiology could be attributed (26.7%) compared to by blood culture alone (8%).

The discordance for *Ureaplasma* spp. between TAC and culture results is largely due to current routine microbiological methods not able to grow *Ureaplasma*. The dominant association of *Ureaplasma* spp., which commonly colonizes the female urogenital tract,[27] and EOS was an unexpected finding in our study. These findings are similar to those reported in ANISA where *Ureaplasma* spp. was attributed as the common cause of EOS.[22] Contrary to our findings where we did not find differences in prevalence of *Ureaplasma* between survivors and non-survivors, ANISA study reported a high prevalence of *Ureaplasma* among non-survivors suggesting pathogenicity and virulence of this organism. *Ureaplasma* spp. has been previously described as a cause of neonatal pneumonia, bacteremia, and meningitis particularly in preterm births.[28] Further studies on the role of *Ureaplasma* in pathogenesis of EOS are needed to determine whether changes to current WHO empiric antibiotic treatment guidelines are warranted.

The use of molecular diagnostics in our study, resulted in a 70% increase in estimated incidence of early-onset invasive GBS disease (1.91/1,000 births) compared to culture-only based incidence (1.12/1,000 births) which was consistent with previous estimates from this site (1.28–1.55/1,000 births).[29] This finding highlights that the current global burden of early-onset disease estimates are likely an under-estimation of the role of GBS in neonatal sepsis when estimated using only blood culture as a diagnostic tool.[30] The discordance noted overall between the traditional blood culture results (8% of cases blood culture positive) and blood PCR-based test, TAC (37% of cases blood TAC positive) is most likely due to TAC detecting culturable and non-culturable organisms (including viruses and *Ureaplasma*). Also, the sensitivity of blood culture could be affected by multiple factors, including volume of blood inoculated, and antibiotic inhibition. TAC also having an advantage of being able to detect both viable and non-viable organisms, thus most likely why some of the healthy neonates had positive TAC results. Compared to NP/OP molecular results, blood TAC tests had higher mean cycle threshold (CT) values, consistent with low levels of pathogen DNA present in the blood. Moreover vastly different sample volumes were evaluated by each test (blood culture: an attempted 1 ml minimum; blood TAC: 300 μL), so the results would not be expected to have strong concordance unless very high genome quantities were present in the blood and all organisms were viable. Furthermore, the background positivity of antigen detection by TAQ in the healthy population, would inherently undermine the expected concordance between blood culture and the TAC readout.

Despite this study increasing our understanding on the causes of neonatal sepsis, the study has several limitations. First, there is no gold-standard case definition for neonatal sepsis potentially leading to case misclassification and limited comparability of study results. This highlights the need to develop more specific clinical criteria for diagnosing neonatal sepsis. To address this we used a case definition that was previously applied in this population, and stratified results by disease severity to increase specificity. Sample collection and blood-culture processing relied upon standard hospital practice, which may not reflect optimized methods of sample collection and laboratory conditions. This is highlighted by finding of an increase in *Pseudomonas aeruginosa* detection in TAC limited to a short period of three months, which we assessed as contamination of the sample after blood was inoculated into the blood culture bottles. Our assessment that this was related to contamination was supported by assessment of the patients showing no difference in severity based on detection of *Pseudomonas aeruginosa*. A limitation of the partial latent class etiologic attribution model [22, 26] is that it does not cater for cases with concurrent multiple pathogens. In this study, it is unlikely that this limitation would have affected the findings, as there were only 7 patients with multiple pathogens out of 99 patients with positive cultures.

Also, an exclusion criteria of our study was perceived imminent death of the newborn, which might have been due to severe infection and consequently we might have under-estimated the burden of invasive disease by excluding them. This was, however, done based on sensitivity issues about approaching the parents for study participation immediately after they were informed of the likely poor prognosis of their newborn. Another limitation of the study is that a number of patients who might have had sepsis were not included, these included babies who were not enrolled because they were born over weekends, or born to mothers with ages <18 years, or were enrolled as they had physician defined sepsis but did not have TAC performed because they did not meet study defined sepsis criteria. Ultimately, the inability to determine an etiology in over three-quarters of cases despite using a combination of tests suggests that significant diagnostic limitations remain or that mucosal infections without bacteremia/viremia or non-infectious processes are responsible for the sepsis-like presentation of a majority of newborns in the first days of life.

In conclusion, results from our study highlight the dominant role of GBS and possible role of *Ureaplasma* spp. in the pathogenesis of EOS. Furthermore, although molecular diagnostic provides an opportunity in improving our understanding on the etiology of EOS, the interpretation of such tests warrant careful analytical approaches. Further studies, including the use of minimal invasive tissue sampling on neonates dying of suspected sepsis is warranted to clarify the causes of neonatal death generally and that attributed to infection.[31]

## Supporting information

S1 Table Clinical and laboratory criteria used to define cases. (DOCX)

S2 Table List of organisms according to whether culturable, tested by Taqman Array Card in blood and respiratory specimens, and those included in etiologic modeling. (DOCX)

S3 Table Observed pathogen detection by Taqman Array Card in neonates with sepsis by case severity*. (DOCX)

S4 Table Observed pathogen detection by Taqman Array Card in neonates with sepsis by infant human immunodeficiency virus infection exposure*. (DOCX)

S5 Table Estimated pathogen proportions stratified by covariates controlling for seasonality. (DOCX)

S1 Text Brief overview of the statistical methodology. (DOCX)

S1 Fig Estimated proportion of total cases caused by each pathogen (pathogen proportion) with 95% confidence intervals stratified by case severity (severe case: infant lethargy noted, NICU admission, death). Legend: *Pathogen proportion estimated indirectly from the “other blood culture” class. Footnote: 68.8% (62.6–73.9%) of severe case episodes and 74.9% (69.3–79.4%) of non-severe episodes could not be attributed to an evaluated pathogen class.
(TIF)
