# Supplementary material for: Surveillance for incidence and etiology of early-onset neonatal sepsis in Soweto, South Africa
Source: PLoS One. 2019 Apr 10;14(4):e0214077. doi: 10.1371/journal.pone.0214077 (PMC6457488; doi:10.1371/journal.pone.0214077)
Supplement: S1 Table — (DOCX) [file pone.0214077.s001.docx]

# S1 Table. Clinical and laboratory criteria used to define cases

| **Clinical Criteria** | **Definition** |
| --- | --- |
| Respiratory instability | respiratory rate >60 breaths per minute; chest wall indrawing; grunting on expiration; respiratory distress noted in medical records, requiring oxygen and/or ventilator support |
| Hypotension | mean arterial pressure <2 standard deviations from mean for weight/age |
| Bradycardia | heart rate <110 beats/min in the absence of beta-blockers or congenital heart disease over a 30 minute period |
| Tachycardia | heart rate >180 beats/ min in the absence of chronotropic drugs and painful stimuli over a 30 minute period |
| Pyrexia or Hypothermia | axillary temperature >38.0°C, or axillary temperature <36.0°C |
| Abdominal/ feeding problems | abdominal distension or feeding intolerance (>20% residual over 24 hours) or poor feeding after have been feeding well, or >2 episodes of emesis |
| Bleeding diathesis | petechiae, ecchymosis, mucous membrane bleeding, or excessive oozing from venepuncture sites |
| Lethargy or irritability | lethargy or irritability noted by medical staff in absence of other central nervous system symptoms |
| Skin and subcutaneous lesions | petechiae, sclerema, mottled skin and prolonged capillary refill time |
| Central nervous system | seizures, or bulging fontanelle, or single witnessed episode of apnoea |
| Metabolic abnormalities | hyperglycaemia (blood glucose >180 mg/dL or 10 mmols/L) or  hypoglycaemia (blood glucose <45 mg/dL or 2.5 mmols/L)  metabolic acidosis: base excess (BE) <-10mEq or serum lactate >2 mmols/L |
| **Laboratory Criteria** |  |
| White blood cell count (WBC) | WBC <5 x 10^9^ /L OR >25 x 10^9^/L |
| Absolute neutrophil count (ANC) | ANC <1.75 x 10^9^/L OR >15 x 10^9^/L |
| Platelet count | <150 x 10^9^/L |
| C- reactive protein | >10.0 mg/L |
| Elevated CSF white cell count (WCC) | >30 x 10^6^ /L WCC in absence of significant red blood cells |

***Footnote:*** Case definitions and criteria adapted from Cutland, et al.^1^

*Sepsis case*: clinical presentation with signs suggestive of sepsis (respiratory instability — one criteria required) or at least two other clinical criteria (table) in the absence of another recognizable congenital infection on days 0–2 of life, and at least one laboratory criteria (table).

*Culture confirmed sepsis*: Isolation of a micro-organism that is not a common contaminant (*Bacillus species,* coagulase-negative staphylococci*, Corynebacterium spp., Micrococcus*) from a normally sterile body site at 0–2 days of life.

^1^Cutland CL, Madhi SA, Zell ER, et al. Chlorhexidine maternal-vaginal and neonate body wipes in sepsis and vertical transmission of pathogenic bacteria in South Africa: a randomised, controlled trial. Lancet. 2009;374(9705):1909-16.
