# Supplementary material for: Surveillance for incidence and etiology of early-onset neonatal sepsis in Soweto, South Africa
Source: PLoS One. 2019 Apr 10;14(4):e0214077. doi: 10.1371/journal.pone.0214077 (PMC6457488; doi:10.1371/journal.pone.0214077)
Supplement: S2 Table — (DOCX) [file pone.0214077.s002.docx]

# S2 Table: List of organisms according to whether culturable, tested by Taqman Array Card in blood and respiratory specimens, and those included in etiologic modeling

| Organism | Culture | Blood  Taqman Array Card | Respiratory  Taqman Array Card | Modeled |
| --- | --- | --- | --- | --- |
| Adenovirus |  |  | X |  |
| *Bordetella pertussis I* |  |  | X |  |
| *Chlamydia pneumoniae* |  |  | X |  |
| *Chlamydia trachomatis* |  |  | X |  |
| Cytomegalovirus |  |  | X | X |
| *E. coli/Shigella* | X | X | X | X |
| Rhinovirus/Enterovirus |  | X | X | X |
| Group A *Streptococcus* | X | X |  |  |
| Group B *Streptococcus* | X | X | X | X |
| *Human* metapneumovirus |  |  | X |  |
| Human parechovirus |  |  | X |  |
| Influenza A |  |  | X |  |
| Influenza B |  |  | X |  |
| *Klebsiella pneumoniae* | X | X | X | X |
| *Mycoplasma pneumoniae* |  |  | X |  |
| *Neisseria meningitidis* | X | X |  | X |
| *pan-Haemophilus influenzae* | X | X |  |  |
| pan-*Salmonella* | X | X |  | X |
| Parainfluenza virus 1 |  |  | X |  |
| Parainfluenza virus 2 |  |  | X |  |
| Parainfluenza virus 3 |  |  | X |  |
| *Pseudomonas aeruginosa* | X | X |  |  |
| Respiratory syncytial virus |  |  | X |  |
| Rubella |  |  | X |  |
| *Staphylococcus aureus* | X | X |  | X |
| *Streptococcus pneumoniae* | X | X | X | X |
| *Ureaplasma* spp. |  | X | X | X |
| *Acinetobacter Baumannii* | X |  |  | X* |
| Viridans streptococci | X |  |  | X* |
| *Enterococcus faecalis* | X |  |  | X* |
| * Estimated indirectly from the “other blood culture” class  Culture: Culturable using the BACTEC automated blood culture system | | | | |
| Blood Taqman Array Card: Organisms included on whole blood TaqMan array card panel | | | | |
| Respiratory Array Card: Organisms included on respiratory (NP/OP) TaqMan array card panel | | | | |
| Modeled: Included in etiologic models based on the Partially Latent Class Model developed by Wu, et al.^1^ | | | | |

^1^Wu Z, Deloria-Knoll M, Zeger SL. Nested partially latent class models for dependent binary data; estimating disease etiology. Biostatistics. 2017;18(2):200-13
