# Supplementary material for: Surveillance for incidence and etiology of early-onset neonatal sepsis in Soweto, South Africa
Source: PLoS One. 2019 Apr 10;14(4):e0214077. doi: 10.1371/journal.pone.0214077 (PMC6457488; doi:10.1371/journal.pone.0214077)
Supplement: S3 Table — (DOCX) [file pone.0214077.s003.docx]

# S3 Table: Observed pathogen detection by Taqman Array Card in neonates with sepsis by case severity*

|  | **Severe Cases** | | **Non-Severe Cases** | | **OR (95% CI)** |
| --- | --- | --- | --- | --- | --- |
| Name | n | % | n | % |  |
| **Blood Specimens: Total** | **238** | | **695** | |  |
| *Ureaplasma* spp. | 19 | 8.0 | 67 | 9.6 | 0.81 (0.5-1.4) |
| Group B *Streptococcus* | 18 | 7.6 | 47 | 6.8 | 1.13 (0.6-2.0) |
| *Pseudomonas aeruginosa* | 1 | 0.5 | 6 | 1.2 | 0.47 (0.01-3.9) |
| *Staphylococcus aureus* | 2 | 0.8 | 10 | 1.4 | 0.58 (0.06-2.8) |
| *E. coli/Shigella* | 8 | 3.4 | 16 | 2.3 | 1.48 (0.5-3.7) |
| *Streptococcus pneumoniae* | 34 | 14.3 | 98 | 14.1 | 1.02 (0.6-1.6) |
| *Klebsiella pneumoniae* | 12 | 5.0 | 23 | 3.3 | 1.55 (0.7-3.3) |
| Pan-*Salmonella* | 11 | 4.6 | 18 | 2.6 | 1.82 (0.8-4.1) |
| *Neisseria meningitidis* | 4 | 1.7 | 6 | 0.9 | 1.96 (0.4-8.4) |
| Enterovirus | 0 | 0.0 | 2 | 0.3 | NA |
| Group A *Streptococcus* | 0 | 0.0 | 2 | 0.3 | NA |
| *pan-Haemophilus influenzae* | 1 | 0.4 | 5 | 0.7 | 0.58 (0.01-5.2) |
| **Respiratory Specimens: Total** | **317** | | **887** | |  |
| *Ureaplasma* spp. | 74 | 23.3 | 166 | 18.7 | 1.32 (1.0-1.8) |
| Group B *Streptococcus* | 38 | 12.0 | 64 | 7.2 | 1.75 (1.1-2.7)** |
| *E. coli/Shigella* | 31 | 9.8 | 74 | 8.3 | 1.19 (0.7-1.9) |
| Enterovirus | 5 | 1.6 | 12 | 1.4 | 1.17 (0.3-3.6) |
| Human metapneumovirus | 1 | 0.3 | 4 | 0.5 | 0.70 (0.01-7.1) |
| *Klebsiella pneumoniae* | 29 | 9.1 | 114 | 12.9 | 0.68 (0.4-1.1) |
| Human parechovirus | 1 | 0.3 | 3 | 0.3 | 0.93 (0.02-11.7) |
| Cytomegalovirus | 21 | 6.6 | 48 | 5.4 | 1.24 (0.7-2.2) |
| *Bordetella pertussis I* | 2 | 0.6 | 4 | 0.5 | 1.40 (0.1-9.8) |
| Rhinovirus | 3 | 0.9 | 5 | 0.6 | 1.68 (0.3-8.7)) |
| *Streptococcus pneumoniae* | 5 | 1.6 | 22 | 2.5 | 0.63 (0.2-1.7) |
| Respiratory syncytial virus | 0 | 0.0 | 3 | 0.3 | NA |
| Adenovirus | 0 | 0.0 | 0 | 0.0 | NA |
| *Chlamydia pneumoniae* | 1 | 0.3 | 1 | 0.1 | 2.80 (0.04-219.9) |
| *Chlamydia trachomatis* | 1 | 0.3 | 5 | 0.6 | 0.56 (0.01-5.0) |
| Influenza A | 0 | 0.0 | 0 | 0.0 | NA |
| Influenza B | 0 | 0.0 | 0 | 0.0 | NA |
| *Mycoplasma pneumoniae* | 0 | 0.0 | 0 | 0.0 | NA |
| Rubella | 2 | 0.6 | 9 | 1.0 | 0.62 (0.06-3.0) |
| Parainfluenza virus 1 | 2 | 0.6 | 2 | 0.2 | 2.81 (0.2-38.9) |
| Parainfluenza virus 2 | 0 | 0.0 | 0 | 0.0 | NA |
| Parainfluenza virus 3 | 0 | 0.0 | 0 | 0.0 | NA |

*Severe case: Infant lethargy noted, or admission to the NICU, or death

** OR (Odds Ratio) significant at p < 0.05; 95% CI (95% confidence interval)
