# Supplementary material for: Surveillance for incidence and etiology of early-onset neonatal sepsis in Soweto, South Africa
Source: PLoS One. 2019 Apr 10;14(4):e0214077. doi: 10.1371/journal.pone.0214077 (PMC6457488; doi:10.1371/journal.pone.0214077)
Supplement: S4 Table — (DOCX) [file pone.0214077.s004.docx]

# S4 Table: Observed pathogen detection by Taqman Array Card in neonates with sepsis by infant human immunodeficiency virus infection exposure*

|  | **HIV Exposure** | | **No HIV Exposure** | | **OR (95% CI)** |
| --- | --- | --- | --- | --- | --- |
| Name | n | % | n | % |  |
| **Blood Specimens: Total** | **296** | | **637** | |  |
| *Ureaplasma* spp. | 31 | 10.5 | 55 | 8.6 | 1.24 (0.8-2.0) |
| Group B *Streptococcus* | 17 | 5.7 | 48 | 7.5 | 0.75 (0.4-1.4) |
| *Pseudomonas aeroginosa* | 1 | 0.4 | 6 | 1.3 | 0.34 (0.01-2.9) |
| *Staphylococcus aureus* | 2 | 0.7 | 10 | 1.6 | 0.43 (0.05-2.0) |
| *E. coli/Shigella* | 4 | 1.4 | 20 | 3.1 | 0.42 (0.1-1.3) |
| *Streptococcus pneumoniae* | 38 | 12.8 | 94 | 14.8 | 0.85 (0.6-1.3) |
| *Klebsiella pneumoniae* | 10 | 3.4 | 25 | 3.9 | 0.86 (0.4-1.9) |
| Pan-*Salmonella* | 11 | 3.7 | 18 | 2.8 | 1.33 (0.6-3.0) |
| *Neisseria meningitidis* | 4 | 1.4 | 6 | 0.9 | 1.44 (0.3-6.1) |
| Enterovirus | 0 | 0.0 | 2 | 0.3 | NA |
| Group A *Streptococcus* | 0 | 0.0 | 2 | 0.3 | NA |
| *pan-Haemophilus influenzae* | 3 | 1.0 | 3 | 0.5 | 2.16 (0.3-16.2) |
| **Respiratory Specimens: Total** | **373** | | **831** | |  |
| *Ureaplasma* spp. | 71 | 19.0 | 169 | 20.3 | 0.92 (0.7-1.3) |
| Group B *Streptococcus* | 24 | 6.4 | 78 | 9.4 | 0.66 (0.4-1.1) |
| *E. coli/Shigella* | 34 | 9.1 | 71 | 8.5 | 1.07 (0.7-1.7) |
| Enterovirus | 8 | 2.1 | 9 | 1.1 | 2.00 (0.7-5.9) |
| *Human metapneumovirus* | 1 | 0.3 | 4 | 0.5 | 0.56 (0.01-5.6) |
| *Klebsiella pneumoniae* | 44 | 11.8 | 99 | 11.9 | 0.99 (0.7-1.5) |
| Human parechovirus | 3 | 0.8 | 1 | 0.1 | 6.72 (0.5-353.0) |
| Cytomegalovirus | 37 | 9.9 | 32 | 3.9 | 2.75 (1.6-4.6)** |
| *Bordetella pertussis I* | 0 | 0.0 | 6 | 0.7 | NA |
| Rhinovirus | 3 | 0.8 | 5 | 0.6 | 1.34 (0.2-6.9) |
| *Streptococcus pneumoniae* | 8 | 2.1 | 19 | 2.3 | 0.94 (0.4-2.3) |
| Respiratory syncytial virus | 0 | 0.0 | 3 | 0.4 | NA |
| Adenovirus | 0 | 0.0 | 0 | 0.0 | NA |
| *Chlamydia pneumoniae* | 1 | 0.3 | 1 | 0.1 | 2.23 (0.03-175.1) |
| *Chlamydia trachomatis* | 2 | 0.5 | 4 | 0.5 | 1.11 (0.1-7.8) |
| Influenza A | 0 | 0.0 | 0 | 0.0 | NA |
| Influenza B | 0 | 0.0 | 0 | 0.0 | NA |
| *Mycoplasma pneumoniae* | 0 | 0.0 | 0 | 0.0 | NA |
| Rubella | 2 | 0.5 | 9 | 1.1 | 0.49 (0.05-2.4) |
| Parainfluenza virus 1 | 0 | 0.0 | 4 | 0.5 | NA |
| Parainfluenza virus 2 | 0 | 0.0 | 0 | 0.0 | NA |
| Parainfluenza virus 3 | 0 | 0.0 | 0 | 0.0 | NA |

* No evidence of maternal HIV versus mother HIV positive (HIV undocumented for 56 case infants)

** OR (Odds Ratio) significant at p < 0.05; 95% CI (95% confidence interval)
