# Supplementary material for: Surveillance for incidence and etiology of early-onset neonatal sepsis in Soweto, South Africa
Source: PLoS One. 2019 Apr 10;14(4):e0214077. doi: 10.1371/journal.pone.0214077 (PMC6457488; doi:10.1371/journal.pone.0214077)
Supplement: S5 Table — (DOCX) [file pone.0214077.s005.docx]

# S5 Table: Estimated pathogen proportions stratified by covariates controlling for seasonality

| **Pathogen** | **All** | **Severe** | **HIV unexposed**** | **HIV exposed** |
| --- | --- | --- | --- | --- |
|  | N = 1,231  %* (95%CI) | N = 332 %* (95%CI) | N = 842  %* (95%CI) | N = 389 %* (95%CI) |
| Group B *Streptococcus* | 4.8 (4.1-5.8) | 7.6 (6.4-9.4) | 5.8 (4.8-6.9) | 2.8 (2.1-4.0) |
| *Ureaplasma* spp. | 5.4 (3.6-8.0) | 5.2 (3.0-9.0) | 5.5 (3.7-8.1) | 5.2 (2.8-9.0) |
| Cytomegalovirus | 1.2 (0.4-2.7) | 1.4 (0.3-3.5) | 1.2 (0.3-2.8) | 1.3 (0.2-4.2) |
| *E. coli/Shigella* | 1.5 (1.1-2.3) | 2.0 (1.3-3.5) | 1.3 (0.8-2.2) | 2.0 (1.4-3.0) |
| *Klebsiella pneumoniae* | 1.8 (0.7-3.7) | 2.3 (0.6-5.2) | 1.7 (0.5-4.0) | 2.1 (0.5-4.6) |
| Pan-*Salmonella* | 1.1 (0.4-2.5) | 1.7 (0.3-4.2) | 0.7 (0.1-2.3) | 2.1 (0.7-4.5) |
| *Staphylococcus aureus* | 1.0 (0.4-2.2) | 0.7 (0.5-1.5) | 1.5 (0.6-3.2) | -- |
| *Neisseria meningitidis* | 0.5 (0.2-1.2) | -- | 0.7 (0.4-1.7) | -- |
| *Streptococcus pneumoniae* | 0.6 (0.4-1.1) | 0.9 (0.5-1.9) | 0.5 (0.3-1.1) | 0.8 (0.4-1.9) |
| Rhinovirus/Enterovirus | 0.2 (0.1-0.3) | -- | 0.2 (0.1-0.5) | -- |
| *Acinetobacter baumannii* | 0.7 (0.6-0.8) | 1.8 (1.5-2.2) | 0.7 (0.6-0.8) | 0.6 (0.5-0.9) |
| *Enterococcus faecalis* | 1.4 (1.2-1.8) | 0.5 (0.4-0.7) | 1.7 (1.3-2.2) | 1.0 (0.7-1.4) |
| *Viridans streptococci* | 4.2 (3.5-5.1) | 4.1 (3.4-5.0) | 4.2 (3.4-5.3) | 4.2 (3.3-5.6) |
| Other culture pathogens | 2.4 (2.0-3.0) | 3.0 (2.3-4.2) | 1.2 (0.9-1.6) | 5.0 (4.0-6.5) |
| Unknown | 73.3 (68.2-77.2) | 68.8 (62.6-73.9) | 73.3 (68.1-77.9) | 73.1 (65.6-78.6) |
| * Pathogen proportion is defined as the percent of total cases in which a given pathogen has played a causative role in disease development | | | | |
| ** Includes 56 case infants with unknown maternal HIV status | | | | |
