# Supplementary material for: Surveillance for incidence and etiology of early-onset neonatal sepsis in Soweto, South Africa
Source: PLoS One. 2019 Apr 10;14(4):e0214077. doi: 10.1371/journal.pone.0214077 (PMC6457488; doi:10.1371/journal.pone.0214077)
Supplement: S1 Text — (DOCX) [file pone.0214077.s006.docx]

# S1 Text: Brief overview of the statistical methodology

1. **The basic partially-Latent Class Model (pLCM):**

SANISA statistical methodology was an extension of the basic partially-Latent Class Model (pLCM) developed by Wu et al for the Pneumonia Etiology Research for Child Health (PERCH) to estimate the proportion of pneumonia infections attributed to specific pathogens.^[1, 2]^ The structure of the basic pLCM used in SANISA can be summarized as below.

Suppose there are $K$ targeted pathogens (as on the TAC cards or isolated by blood culture in SANISA) each with one diagnostic test $T_{k}, k=1,\ldots K$ that produces binary (positive vs negative, or 1 vs. 0) test result $y_{ik}$ for case$i, i=1,\ldots,N$. We add one extra class (referred to throughout as ‘Other/None’) for other pathogenic or non-pathogenic causes and code it as class$(K+1)$. If we assume each individual case has only one etiology cause from the $K+1$ classes, then the population of cases can be considered as a mixture of subpopulations with etiology$k=1,\ldots,K, K+1$. Let $Z_{i}$ be the true (unobserved) etiology of case$i, i=1,\ldots,N$, then the objective of the model is to estimate probability$\pi_{k}=P\left( Z_{i}=k \right), k=1,2,\ldots,K,K+1$, using the observed binary test results$\left\{ y_{ik}, k=1,\ldots,K;i=1,\ldots,N \right\}$. Here $0\leq\pi_{k}\leq1$ and$\sum_{k=1}^{K+1} \pi_{k}=1$. We refer to $\pi_{k}$ as pathogen proportion throughout.

pLCM expresses the probability of observing $\left\{ y_{ik} \right\}$ through a linear mixture model with ${\{\pi}_{k}\}$ as the mixing coefficients. By applying the regular conditional independence assumption for such linear mixture class models with unknown (latent) classes, and a further assumption that the probability of test $T_{k}$ to produce positive test result depends only on whether pathogen $k$ is the true etiology of the tested case, the linear mixture can be simplified as:

$f\left( y_{ik}, k=1,\ldots,K;i=1,\ldots,N \right)= \prod_{i=1}^{n} (\sum_{k=1}^{K} \pi_{k}\theta_{k}^{y_{ik}}\left( 1-\theta_{k} \right)^{1-y_{ik}}\prod_{j\neq k} \delta_{j}^{y_{ij}}\left( 1-\delta_{j} \right)^{1-y_{ij}}+ \pi_{K+1}\prod_{j} \delta_{j}^{y_{ij}}\left( 1-\delta_{j} \right)^{1-y_{ij}})$ [1]

Here parameters $\theta_{k}=f\left( y_{ik}=1 \right| true etiology=k)$ and $\delta_{k}=f\left( y_{ik}=1 \right| true etiology \neq k)$ are called the True Positive Rate (TPR) and False Positive Rate (FPR) respectively for test$T_{k}, k=1,\ldots K$.

The parameters in Equation 1 can be estimated under a Bayesian analysis framework using conjugate priors for the parameters, for example, (K+1)-class Dirichlet distributions for the pathogen proportions and Beta distributions for the TPRs and FPRs. With minor modifications, the basic pLCM model can be extended to situations where multiple pathogen-specific tests are performed, including blood culture. Additional pathogen classes could be created to account for pathogen co-infections.^[1]^

Deloria-Knoll et al demonstrated that pLCM outperforms the regular etiology fraction method based on population attributable risk (PAR).^[3]^ Further, they showed that collection of specimens from a large sample of healthy infants (providing initial estimates of FPRs), inclusion of an adequate number of cases with confirmed pathogen-specific infections (such as through blood culture isolation), and prior knowledge of the TPRs associated with diagnostic tests used for a subset of pathogens, allow the basic pLCM to produce reliable pathogen proportion estimates. Our simulation experiments also revealed a few situations where pLCM might perform less well, notably when pathogens only had a single laboratory test performed, true pathogen proportions were very low and either FPR values were high or TPR and FPR were close in value. In SANISA, we developed mitigation strategies (see Section 3) to avoid inclusion of such pathogens in the pLCM, and where mitigation was not possible, we excluded such pathogens by combining them into the “Other/None” class.

1. **An extension of pLCM with covariate dependent pathogen proportions and false positive rates**

The TAC tests employed in SANISA were developed by extensive testing of the target pathogens and nearest neighbors to ensure high laboratory specificity. Hence, a positive test result almost certainly indicated that the pathogen was present in the collected specimen. Since we assume a single etiology cause for each case, positives for non-etiological pathogens indicate pathogen carriage. Because pathogen carriage rates likely change over many covariates such as HIV status and season (enrollment date), false positive rates in pLCM should vary similarly. However, the assignment of pathogen classes as the cause of a pSBI episode is established by comparing the true and false positive rates. If false positive rates vary by covariates, an identical set of test results may have different etiologic meaning at different covariate values. Consequently, pathogen proportions cannot be determined by test results alone but should also vary by covariates. Thus, we extended the basic pLCM to the following model:

$f\left( \tilde{Y}_{1}, \cdots, \tilde{Y}_{n} \right)= \prod_{i=1}^{n} (\sum_{k=1}^{K} \pi_{k}^{x_{i}}\theta_{k}^{y_{ik}}\left( 1-\theta_{k} \right)^{1-y_{ik}}\prod_{l\neq k} {(\delta_{l}^{x_{i}})}^{y_{il}}\left( 1- \delta_{l}^{x_{i}} \right)^{1-y_{il}}+ \pi_{K+1}^{x_{i}}\prod_{l} {(\delta_{l}^{x_{i}})}^{y_{il}}\left( 1- \delta_{l}^{x_{i}} \right)^{1-y_{il}})$ [2]

Here $(x_{1},\ldots,x_{n})$ are the observed values of the covariates for the *n* cases. $\tilde{\Pi}^{x}=\left( \pi_{1}^{x}, \cdots,\pi_{K}^{x},\pi_{K+1}^{x} \right)$ and $\tilde{\Delta}^{x}=\left( \delta_{1}^{x}, \cdots,\delta_{K}^{x} \right)$ are the pathogen proportion distributions and false positive rates for any $x \in\mathcal{X}$. Notice that we hold the true positive rates $\Theta=\left( \theta_{1},\ldots,\theta_{K} \right)$ constant across covariate levels because we assume that infection by a pathogen implies presence of the pathogen in the respiratory or blood samples from cases.

Allowing false positive rates to vary by covariates is not only necessary to address known variation in pathogen carriage, but also alleviates violations of the conditional independence assumption in pLCM. This is because covariates are often confounders for co-carriage of some pathogens. Through adjusting the confounding effect, dependence between test results for co-carried pathogens may weaken, both locally and globally.

A Bayesian Kernel Model approach was developed to estimate parameters in the extended model as expressed by Equation 2. For each data point $x$ in the domain of the covariates, we assume pathogen proportion $\tilde{\Pi}^{x}=\left( \pi_{1}^{x}, \cdots,\pi_{K}^{x},\pi_{K+1}^{x} \right)$ to have a Dirchlet prior with parameters$\left( e_{1}^{x0}, \cdots,e_{K}^{x0},e_{K+1}^{x0} \right)$, and the false positive rates $\delta_{k}^{x}, k=1,\ldots,K$ to have Beta priors with parameters$\left( c_{k}^{x0},d_{k}^{x0} \right),k=1,\ldots,K$. We also assume the posterior distributions of the parameters can be approximated by the same type of distributions and thus use them as the sampling distributions in the next iteration of the Gibbs Sampler. The parameters of the sampling distributions are updated by the following equations:

$e_{k}^{x}=e_{k}^{x0}+\sum_{i=1}^{N} z_{ik}\times d(x,x_{i}), k=1,\ldots,K, K+1$ [3]

$c_{k}^{x}=c_{k}^{x0}+\sum_{i=1}^{N} y_{ik}\times z_{i\left( K+1 \right)} \times d(x,x_{i}), k=1,\ldots,K$ [4a]

$d_{k}^{x}=d_{k}^{x0}+\sum_{i=1}^{N} {(1-y}_{ik})\times z_{i\left( K+1 \right)} \times d(x,x_{i}), k=1,\ldots,K$ [4b]

Here $Z_{i}= \left( z_{i1},z_{i2},\ldots,z_{iK},z_{i(K+1)} \right)$are the imputed pathogen (latent) classes for the etiology of case *i* from the previous iteration of the Gibbs sampler. $z_{ik}$ takes value 0 or 1 only, and $\sum_{k=1}^{K+1} z_{ik}=1$. The quantity $d\left( x, x_{i} \right)$ measures the contribution of case *i* at $x_{i}$ to data point$x$, such that$0\leq d\left( x, x_{i} \right)\leq1$, $\max_{x} d\left( x, x_{i} \right)=d\left( x_{i},x_{i} \right)=1$, and $d\left( x, x_{i} \right)$ decreases as the distance between $x$ and $x_{i}$ increases. For discrete covariates, $d\left( x, x_{i} \right)$ takes value 1 or 0 depending whether $x_{i}$ and $x$ share the same covariate values. For continuous covariates

$d\left( x,x_{i} \right)=C \times K\left( x \right|x_{i}, h)$ [5]

Here $K\left( x \right|x_{i}, h)$ is a Gaussian density function with mean at $x_{i}$and standard deviation$h$. $C$is a constant to make sure that $d\left( x_{i},x_{i} \right)=1$.

The prior distributions of parameters are constructed by adding (K+1) pseudo cases to the study population (one pseudo case per pathogen class). Each pseudo case has equal probability to be positive or negative for each of the laboratory tests. Hence, the prior distribution for the true positive rates, which are invariant by covariates, are the Jeffery non-informative prior for binary events, or Beta(0.5, 0.5). When covariates are considered, each pseudo case will be considered as a probability density function uniformly distributed on the domain $\mathcal{X}$ of the covariates. Its overall contribution to a data point $x\in\mathcal{X}$ is then:

$d\left( x \right)= \frac{1}{\left| \mathcal{X} \right|}\int_{t \in\mathcal{X}} d\left( x, t \right)dt$ [6]

Here we assume the domain of the covariates is finite and $\left| \mathcal{X} \right|$ is its volume (area). Hence, the prior for the pathogen distribution at data point $x$ will be $Dirichlet(d\left( x \right), d\left( x \right), \ldots, d\left( x \right))$. The overall contribution of the prior distributions to our analyses (= (K+1) / N ) is very small if the number of targeted pathogen K is much smaller than the number of cases N.

The prior distribution for false positive rates is constructed from the data from infants without the disease under study, by calculating contributions of their data points to $x\in\mathcal{X}$, using the same function $d\left( x,y \right)$. For a particular test $T$, we add the contributions from positive and negative results as the Beta parameters for the false positive rate.

The smoothing parameter $h$ in Equation [5] controls the amount of local smoothing. It can be decomposed into two components: $h= \sqrt{h_{c}^{2}+h_{d}^{2}}$. Here $h_{d}$ controls the smoothness of the estimated probability density of the covariates. We use the “rule of thumb” in the density estimation literature to set $h_{d}$. For example, if N is the sample size of cases and there is one continuous covariate, then $h_{d}=N^{-1/5}$ after the covariates are scaled with mean 0.0 and variance 1.0. $h_{c}$ is used to define the neighborhood of data points. We applied knowledge of the epidemiology of the disease under evaluation to narrow the range of the parameters.

1. **Implementation details of the extended pLCM model**

Two strategies were implemented to mitigate inclusion of pathogens with characteristics that might result in unreliable model performance based on simulation experiments (see Section 1). First, we prescreened pathogen lists within each HIV exposure category and each case severity category using a stepwise procedure that excluded pathogens with very few positive TAC tests results. The second strategy flagged remaining pathogens with: a) high false positive rates among infants without sepsis; b) lower positivity rate among cases than among infants without sepsis; and c) significant and substantial pairwise correlations of tests results among infants without sepsis.

The effects of the covariates on flagged pathogens were further examined through stratifications and/or regression models. If the covariates did not reduce the flagged features at least locally, then flagged pathogens were considered for exclusion from the model (no pathogens in SANISA fell in this category). If the flagged features disappeared in some local regions of covariates, but remained in others, then the pathogen was kept in the model, but local estimations in the troubled regions should be interpreted with caution. If a pathogen was not selected in any of the strata, the pathogen effectively was captured by the “Other/None” class. If a pathogen was included in some strata, but not others, then the pathogen proportion was set to zero in the strata where the pathogen was excluded from the target list; because this occurred only for pathogens with very few or no positive test results, setting to zero was the best approximation.

In SANISA, non-informative priors were used for all parameters. The contribution of the priors was equivalent to adding one pseudo case per pathogen class into the case population, or 28 pseudo cases to a cohort of 1,231 protocol-defined cases. Thus, the contribution of priors was so small that our results can be considered as data driven, rather than prior distribution driven. Additionally, in SANISA we set lower limits for TAC test true positive rates: 40% for respiratory TAC and 20% for Blood TAC tests. We did not set a lower limit for blood culture true positive rates.

The neighborhood smoothing parameter $h_{c}$for the continuous variable of enrollment time was chosen as 2.0 months. With this choice of smoothing parameter, the average local sample size, defined as $N_{x,h}= \sum_{i=1}^{N} d(x,x_{i})$, was approximately 276 HIV unexposed cases and 133 HIV exposed cases (roughly one third of the 828 HIV unexposed and 399 HIV exposed cases enrolled). In other words, instead of using 1,231 cases to estimate covariate independent pathogen proportions and false positive rates, the extended pLCM uses an average of 276 and 133 local cases for corresponding HIV unexposed and HIV exposed cases to estimate local model parameters. The local sample sizes appeared capable of capturing major seasonal trends, without creating unstable local random fluctuations.

When updating distributions for false positive rates in Equation [4a] and [4b], only cases assigned to class (K+1), or the class of “Other/None”, were included in order to minimize a concern that infection by a pathogen might change carriage rates of other pathogens.

After a 50,000 iteration burn-in period we ran the ANISA pLCM for 150,000 iterations. Posterior means and the corresponding 95.0% credible intervals of model parameters were then generated from the iterations, either globally or stratified by covariates, or even individually for cases. Continuous curves can also be constructed to visually reveal seasonal patterns of etiology proportions for individual pathogens.

The proportions of pathogens that were isolated by blood culture but not on the target list of TAC cards could not be estimated directly by the pLCM model. We combined them into a pathogen class called “Other Blood Culture”. The proportion of episodes attributed to this combined class was estimated indirectly by calculating the product of the number of blood culture isolates in this class and the average estimated proportion attributed to pathogens with multiple tests that included blood culture and that yielded at least one isolate.

Because the primary output from the model is pathogen proportion, incidence rates (per 1000 live births), were calculated by the product of the total cases and the pathogen-specific proportions divided by total registered live births.

To generate the 150,000 iterations of Gibbs sampler, a total computation time of 30 hours was required (< 1 second per iteration). The SANISA computation program was written in R and can be shared upon request.

**References**

1. Wu Z, Deloria-Knoll M, Zeger SL. Nested partially latent class models for dependent binary data; estimating disease etiology. Biostatistics. 2017;18(2):200-213. DOI: 10.1093/biostatistics/kxw037.

2. O'Brien KL, Baggett HC, Brooks WA, Feikin DR, Hammitt LL, Howie SRC, et al. Introduction to the Epidemiologic Considerations, Analytic Methods, and Foundational Results From the Pneumonia Etiology Research for Child Health Study. Clin Infect Dis. 2017;64(suppl_3):S179-S184. DOI: 10.1093/cid/cix142.

3. Deloria Knoll M, Fu W, Shi Q, Prosperi C, Wu Z, Hammitt LL, et al. Bayesian Estimation of Pneumonia Etiology: Epidemiologic Considerations and Applications to the Pneumonia Etiology Research for Child Health Study. Clin Infect Dis. 2017;64(suppl_3):S213-S227. DOI: 10.1093/cid/cix144.
